# Socioeconomic Status and Psychosomatic Health in Older Adults: Evidence from the Greek Population

**DOI:** 10.1192/j.eurpsy.2025.1759

**Published:** 2025-08-26

**Authors:** F. Tatsis, D. Mazetas, K. Karamanis, M. Gouva

**Affiliations:** 1 Faculty of Medicine; 2 Research Laboratory Psychology of Patients, Families & Health Professionals, Department of Nursing; 3 Department of Accounting and Finance, University of Ioannina, Ioannina, Greece

## Abstract

**Introduction:**

Previous research suggests that individuals with lower socioeconomic status are frequently exposed to more health risks and emotionally stressful conditions compared to those with higher status. There is a clear link between income and poor quality of life, with disadvantaged older adults lacking key resources. This study explores the relationship between the economic profile of older individuals and their psychosomatic health within the Greek population. It continues a previous investigation on the impact of socioeconomic status on health, offering a deeper understanding of long-term effects.

**Objectives:**

This study investigates how socioeconomic status affects the physical and mental health of older adults in Greece, focusing on income levels’ influence on health quality and disparities in health outcomes.

**Methods:**

This cross-sectional study was conducted among 516 Greek participants (214 men, 302 women, average age 73.47 ± 7.72). The participants’ average monthly income was €796.72 ± 621.82 (p=0.002). Psychosomatic health was evaluated using the Other As Shamer Scale (OAS), Experience of Shame Scale (ESS), and Cardiac Anxiety Questionnaire (CAQ). A one-way MANOVA analyzed the impact of income on health quality, focusing on both physical and mental health using the 36-Item Short Form Health Survey (SF-36).

**Results:**

Our findings revealed that individuals with a monthly income above €1,500 reported significantly higher quality of life scores. In contrast, those with lower economic profiles exhibited increased levels of shame (measured by OAS and ESS), anxiety about their heart health (measured by CAQ), and a tendency to somatize stress. The analysis indicated that for every additional €100 of monthly income, physical health quality increased by 4.4 points on the SF-36 Physical Health Scale, and mental health quality improved by 2.9 points on the SF-36 Mental Health Scale. These results underscore the statistically significant impact of monthly income on overall physical and mental health scores.

**Conclusions:**

The study highlights a strong link between lower economic status and poorer physical and mental health among older Greek adults. Addressing these effects requires political and societal efforts, with mental health policies that focus on economically vulnerable populations to mitigate the health impacts of low income and improve overall well-being.

**Disclosure of Interest:**

None Declared

